# The return from underperformance to sustainable world-class level: A case study of a male cross-country skier

**DOI:** 10.3389/fphys.2022.1089867

**Published:** 2023-01-09

**Authors:** Rune Kjøsen Talsnes, Einar Flaktveit Moxnes, Trond Nystad, Øyvind Sandbakk

**Affiliations:** ^1^ Department of Sports Science and Physical Education, Nord University, Bodø, Norway; ^2^ Meråker High School, Trøndelag County Council, Steinkjer, Norway; ^3^ Centre for Elite Sports Research, Department of Neuromedicine and Movement Science, Norwegian University of Science and Technology, Trondheim, Norway

**Keywords:** endurance training, non-functional overreaching, overtraining syndrome, performance development, relative energy deficiency in sports, training load, XC skiing

## Abstract

**Purpose:** To determine the main factors associated with unexpected underperformance and prospectively describe the holistic process of returning to sustainable world-class level in a male cross-country skier.

**Methods:** Longitudinal training data was retrospectively analyzed across nine seasons (2012-2013 to 2020-2021), and categorized into training forms (endurance, strength, and speed), intensities [low- (LIT), moderate- (MIT), and high-intensity training (HIT)], and modes (specific and non-specific). Performance data was obtained from the International Ski and Snowboard Federation. Following two seasons of unexpected underperformance (2019-2020 and 2020-2021), the participant was prospectively followed in the process of returning to sustainable world-class level (2021-2022). Day-to-day training data and physiological tests were analyzed, and interviews with the participant and the head coach conducted.

**Results:** Longitudinal training data from 2012-2013 to 2018-2019 demonstrated a non-linear 30% increase in total training volume (from 772 to 1,002 h), mainly caused by increased volume of ski-specific endurance training without changes in intensity distribution. Coincidingly, the participant gradually reached a world-class performance level. After two seasons of unexpected underperformance with relatively similar training volumes and intensity distributions as in the preceding seasons, the possible contributing factors were identified: lack of training periodization, limited monitoring and intensity control, particularly in connection with a “extreme” regime of training with low carbohydrate availability and days including two MIT sessions, as well as lack of systematic technique training and follow-up by coaches on a daily basis. Consequently, the return to world-class level included the introduction of a clear micro-cycle periodization, more systematic physiological monitoring and testing, more accurate intensity control, increased carbohydrate intake during and between sessions, as well as increased emphasize on technique training and an assistant coach present during day-to-day training.

**Conclusion:** These longitudinal data describe the main factors leading to unexpected underperformance, in addition to providing unique insights into the corresponding process of returning to sustainable world-class level in a male cross-country skier. The holistic approach described in this case study may serve as a theoretical framework for future studies and practical work with underperforming endurance athletes.

## Introduction

Cross-country skiing is a demanding endurance sport challenging the upper limits of human physiology by involving whole-body work of different durations and intensities, often in cold environmental conditions (Holmberg, 2015). Retrospective studies of world-class cross-country skiers have demonstrated training volumes of ∼750–1,000 h annually with the majority being endurance training (∼600–900 h), and the remaining speed and strength training (∼75–100 h) (Sandbakk et al., 2011; Tonnessen et al., 2015; Sandbakk et al., 2016; Sandbakk and Holmberg, 2017; Solli et al., 2017). Herein, most of the endurance training are performed as low-intensity training [LIT, (∼80% of sessions)] with the remaining ∼20% of sessions (∼2-3 sessions per week) are typically performed as moderate-intensity (MIT) and high-intensity training (HIT) (Sandbakk and Holmberg, 2017). While the highest training volumes and largest distributions of LIT is performed during the *general preparation period*, the *specific preparation* and *competition period* are often characterized by more intensification through increased emphasize on HIT sessions (Tønnessen et al., 2014). Although different pathways to success in endurance sports have been described, longitudinal case-studies of world-class endurance athletes (Stellingwerf, 2012; Bourgois et al., 2014; Pinot and Grappe, 2015; Rasdal et al., 2018; Schmitt et al., 2020) including the worlds most decorated female cross-country skier (Solli et al., 2017) suggests the use of a non-linear year-to-year progression in training volume in the process of reaching a world-class level. However, while the training characteristics of successful cross-country skiers are well-described in the scientific literature (Sandbakk and Holmberg, 2017), there are still limited longitudinal data on the development process leading to world-class performance levels.

The high training loads of endurance athletes requires an adequate balance between training and recovery to maximize adaptations while sustaining good health and mitigating side-effects such as maladaptation, fatigue, and injury. In this context, training periodization including different ordering of the training stimulus within micro- and meso-cycles are considered crucial for achieving sufficient training variation, detecting inadequate training loads, and thereby optimizing long-term performance development (Issurin, 2010). In cases of imbalance between stress and recovery, a state of underperformance (i.e., unexpected reduction in performance level) may occur, and manifest into a continuum reaching from functional overreaching (FOR) to non-functional overreaching (NFOR), and the overtraining syndrome (OTS) (Meeusen et al., 2013). OTS can be defined as an accumulation of training and/or non-training stressors resulting in prolonged underperformance with a complex etiology involving a wide range of psychological, biochemical, immunological, and neuroendocrine symptoms (Meeusen et al., 2013). Unexpected underperformance have been suggested to affect 20%–60% of athletes throughout their career (Morgan et al., 1987; Kenttä et al., 2001; Matos et al., 2011; Meeusen et al., 2013), although full-blown OTS diagnosis in elite populations are relatively rare (Buyse et al., 2019).

Adequate nutrition and energy intake to meet the energy expenditure associated with the high training volumes in endurance athletes is of high importance, posing a risk for under-fueling, low-energy availability, and possible diagnosis of relative energy deficiency in sports (RED-S) (Mountjoy et al., 2015; Stellingwerff et al., 2021). RED-S describes a mismatch between energy intake and the energy expended during exercise and have the potential to impair a wide range of body systems (Mountjoy et al., 2015; Stellingwerff et al., 2021). It has recently been addressed that OTS and RED-S have many shared pathways and symptoms initiated from a hypothalamic–pituitary dysfunction that can be influenced by low energy and/or low carbohydrate (CHO) availability (Stellingwerff et al., 2021). The role of low CHO availability (with or without the diagnosis of OTS/RED-S) have been linked to increased risk of developing NFOR and/or OTS in endurance athletes (Meeusen et al., 2013; Heikura et al., 2019; Stellingwerff et al., 2021). Interestingly, training with low CHO availability (i.e., “train low”) have been suggested as a method to augment endurance adaptations (Burke, 2010; Bartlett et al., 2015). However, while training with low CHO availability may induce positive molecular signaling, translation into actual performance adaptations seems limited (Gejl and Nybo, 2021), and may concurrently increase the risk of developing RED-S and/or OTS without careful periodization and monitoring (Jeukendrup, 2017; Stellingwerff et al., 2021).

There is no consensus in the scientific literature on how prolonged underperformance (with or without the diagnosis of OTS and/or RED-S) in endurance athletes should be “treated”. However, the most obvious interventions for athletes in a NFOR or OTS state are modifications of training load combined with adequate recovery (e.g., altering sleep, nutrition, and non-training stressors) (Foster, 1998; Gustafsson et al., 2008; Meeusen et al., 2013). In cases of RED-S diagnosis, adequate treatment involves restoration of energy availability through nutritional interventions, but also potentially reducing energy expenditure through adjustments in training load (Mountjoy et al., 2015; Stellingwerff et al., 2021). However, each case should likely be treated differently, dependent on the reasons for underperformance. Interestingly, a recent case study on the most decorated winter Olympian described the holistic process of returning to world-class level in cross-country skiing following long-term underperformance (Solli et al., 2020). Here, analyses of possible contributing factors to the underperformance determined the subsequent interventions. In this case, increased emphasize on LIT and MIT with a corresponding decrease in HIT, increased autonomy in the training process, reduced non-training stressors, more systematic physiological testing, mental training, altered nutritional strategies, and optimized asthma treatment were the main factors associated with her return to world-class level (Solli et al., 2020). Taken together, the study by (Solli et al., 2020) and a comparable case study on a junior cross-country skier (Gustafsson et al., 2008) underpins the importance of a holistic and individualized approach in “treating” prolonged underperformance in endurance athletes. However, these studies have only retrospectively investigated the process of returning from underperformance, whereas prospective designs of such processes are currently lacking.

Therefore, the purpose of this case study was to determine the main factors associated with unexpected underperformance and to prospectively describe the holistic approach of returning to sustainable world-class level in a male cross-country skier.

## Materials and methods

### Participant

The participant was a 31-year-old Norwegian cross-country skier. In total, the participant has 125 world-cup starts including 4 wins and 14 places at the podium, as well as both Olympic and World champion gold medals in the team relay event. Accordingly, the participant was classified as world-class (Tier 5) according to the classification framework in sport and exercise science developed by (McKay et al., 2021). Moreover, the participant has achieved both a win and podium place in the National cross-country championship and participated in the European cross-country championship in running. The participants anthropometrical and physiological characteristics were: body mass, 71.0 kg; body height, 179.0 cm; body mass index, 22.2 kg m^−2^; maximal oxygen uptake (VO_2max_) in roller-ski skating (G3 sub-technique), 5.59 L min^−1^ and 79.9 mL min^−1^·kg^−1^. The study followed the institutional requirements and was pre-approved by the Norwegian Centre for Research Data. Prior to the study, the participant provided a written informed consent to participate.

### Overall design

The present case study constitutes two parts: 1) retrospective description of longitudinal training data across nine seasons (2012-2013 to 2020-2021) including possible causes of two seasons (2019-2020 and 2020-2021) with unexpected underperformance, and 2) prospective description of the holistic process of returning to sustainable world-class level (2021-2022 season). During this process, day-to-day training data and physiological tests were analyzed, and interviews with the participant and the head coach conducted. The support (sport science) team consisted of a PhD student in sport science and exercise physiologist at the Norwegian Top Sport Centre (first author), a master student in exercise physiology and assistant coach (second author), a head coach and leader of the support team with extensive coaching experience (third author), and a professor in sport science (last author).

### Analyses

All analyses were carried out using Office Excel 2016 (Microsoft Corporation, Redmond, WA, United States). Performance data was obtained for each season using the International Ski and Snowboard Federation (FIS) distance point system and competition results, including World-cups, Olympics, World championships, and National championships (FIS, 2022).

### Training data

The participant recorded his training data using online training diaries. All training was categorized into training forms (endurance, strength, and speed), intensities (LIT, MIT, and HIT), and modes [specific (skiing and roller-skiing) and non-specific (running, cycling, and ski mountaineering)]. The 3-zone endurance intensity scale included the following physiological boundaries: [LIT, <1.5 mmol L^−1^ blood lactate and 55%–82% of maximal heart rate (HR_max_)], MIT, ∼1.5–3.5 mmol L^−1^ blood lactate and 82%–87% of HR_max_) and HIT, >3.5 mmol L^−1^ blood lactate and >87% of HR_max_. Endurance training sessions were registered using the *modified session-goal approach* (Sylta et al., 2014) and included regular use of heart rate recordings and blood lactate measurements. Based on these intensity measures, the participant allocated the different parts of the sessions into the respective intensity zones. Interval training sessions were recorded as total time of all intervals combined excluding recovery periods. Strength and speed training were recorded from the start to the finish of that specific part during sessions (Solli et al., 2017). Endurance training load was calculated using the training impulse (TRIMP) method by multiplying each minute with a weighing factor (1, 2, and 3 for LIT, MIT and HIT, respectively) as previously described (Foster et al., 2001; Solli et al., 2019). For the seasons 2019-2020 to 2021-2020, the annual training was categorized into different periodization phases: *general preparation period*, *specific preparation period*, and *competition period* according to previous studies (Tønnessen et al., 2014; Solli et al., 2017). In addition, a *periodization index* was developed to better understand differences in training periodization between the seasons with underperformance and the season returning to world-class level. This was achieved by calculating the ratio between the highest and lowest micro-cycle load (1 week) within each meso-cycle (3 weeks). The use of week-to-week comparisons when adopting the *performance index* to this study was done after confirming that the participant had planned his micro-cycles from Monday-to-Sunday during the entire period. The micro-cycles consisting of artificial low loads due to sickness were filtered out and not included in the calculations. There was some missing data in March and April during some seasons which was retrospectively completed by interviews with the participant and the use of training plans and online platforms connected to the heart rate monitors used.

### Physiological testing

The participant underwent regular physiological testing at the Norwegian Top Sport Centre, but due to inconsistency in the test protocols adopted across the seasons investigated, only blood lactate profiling in running for the prospective part (2021-2022 season) could be included in the study. The tests were performed on a motor-driven treadmill in the laboratory using protocols previously described by (Ingjer, 1992; Tønnessen et al., 2015). Speed, heart rate, and rating of perceived exertion at 4 mmol L^−1^ (_@4mmol_) blood lactate were calculated by linear interpolation. The participant also reported the perceived “motivation” and “readiness” prior to each test on a scale ranging from 1 (poor) to 10 (excellent). Moreover, the participant underwent tests of continuous blood glucose monitoring, resting metabolic rate, bone mineral density, and relevant blood markers during these seasons that are not presented in detail here.

### Interviews

Separate interviews with specific questions regarding the seasons of underperformance and the return to world-class level were conducted with the participant and the head coach (leader of the support team) two to three times each. A semi-structured approach was adopted using an identical set of questions, but the ordering of questions differed depending on the corresponding responses, in which sometimes were further explored by the interviewer. The interviews were conducted face-to-face and tape-recorded (30–45 min duration). Further, a content analyses was conducted independently by two of the researchers (EM and RT) to categorize the responses. The answers were categorized into four topics (training, nutritional, physiological, and technical), as presented in the results section. The technical aspects described are based on the participants (self-perceived) and his coach’s (expert opinion) perspectives. Considering that the head coach interviewed and the two researchers performing the content analyses also were a part of the support team, the researchers have carefully pursued to verify and validate the analyses and provided critical interpretations of the data. While this provided unique insight, the reader should be aware of this lack of independency when interpreting the findings of the present case study.

## Results

### Longitudinal training data leading to world-class level

**FIGURE 1:**
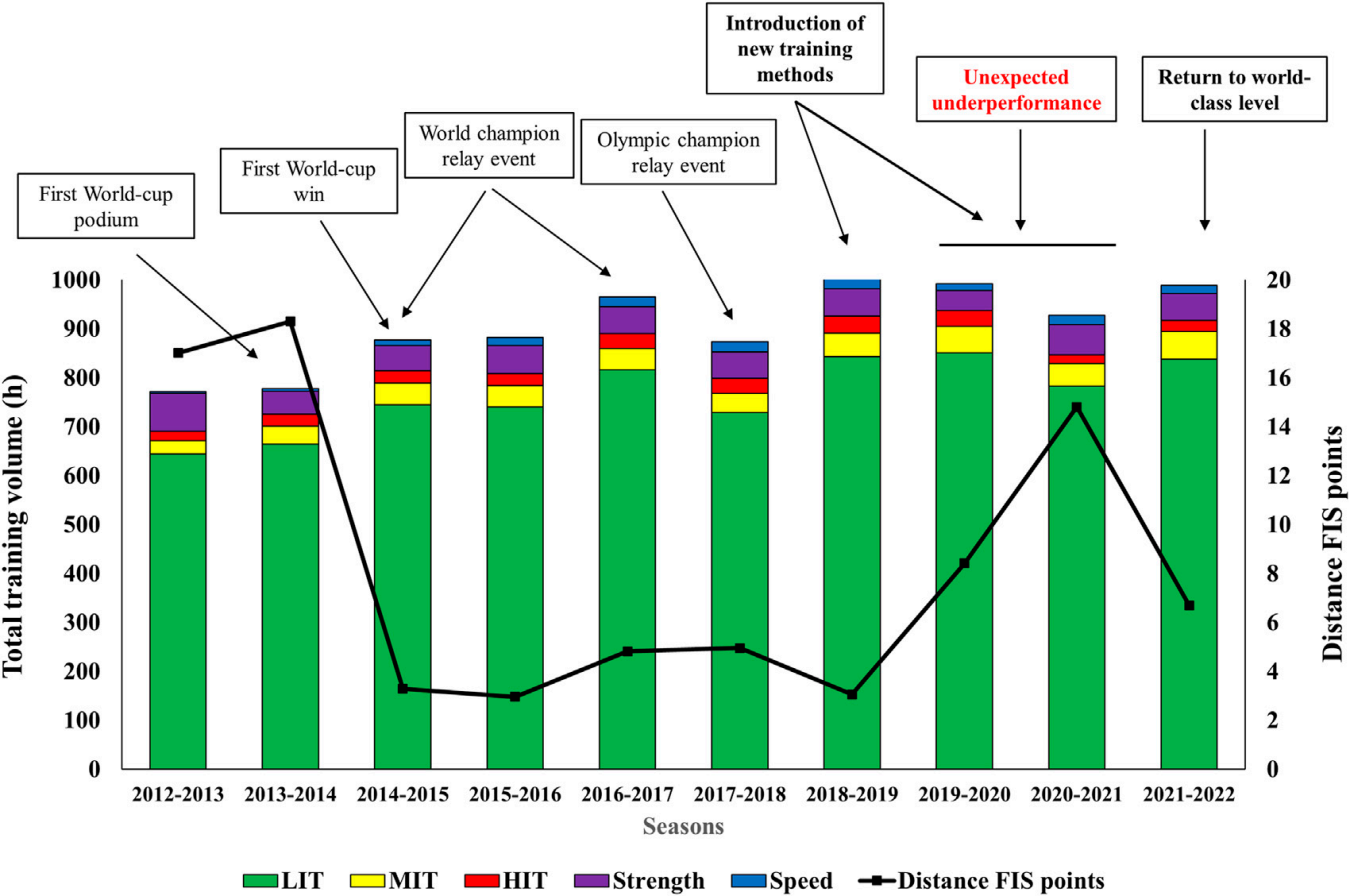
Longitudinal training data and corresponding performance changes (distance FIS points) across ten seasons including two seasons of unexpected underperformance and the season returning to world-class level in a male cross-country skier. LIT indicates low-intensity training; MIT, moderate-intensity training; HIT, high-intensity training.

Longitudinal training and performance data for the 10 seasons investigated are displayed in Figure 1. From the 2012-2013 to the 2018-2019 season, there was a 30% increase in total training volume (from 772 h to 1,002 h). The number of sessions increased by 16% (from 488 to 553). The endurance training volume increased by 34% (from 691 to 926 h) constituting increases of 31% LIT (from 644 to 843 h), 75% MIT (from 28 to 48 h), and 84% HIT (from 19 to 35 h). However, the training intensity distribution remained relatively similar across all seasons (∼91–93% LIT, ∼4-5% MIT, and ∼3-4% HIT). The volume of non-specific endurance training (mainly running) was relatively similar across all seasons (∼250 h) while the volume of specific endurance training (skiing and roller-skiing) increased by 51% (from 440 to 662 h). Moreover, the volume of strength training remained relatively similar (∼55 h), whereas the volume of speed training increased by more than five-fold (from 3 to 20 h). Coincidingly, the participant gradually reached a world-class level as illustrated by the reduction in distance FIS points (Figure 1). From the 2016-2017 to 2018-2019 season, the participant started to experience periods of glycogen depletion during training and competitions (“hitting the wall”), as well as a feeling of low energy availability, and particularly during the nights. This was coincided by increased CHO oxidation during rest, as well as increased body mass (from ∼70 to ∼75 kg) and body fat percentage (from ∼12%–17%).

### Factors associated with unexpected underperformance

During the 2019-2020 season, the participant started to experience periods of unexpected underperformance that accumulated in the subsequent 2020-2021 season, resulting in that the participant ended the season already in January. Although the total training volume and intensity distribution during these two seasons remained relatively similar as in the preceding seasons (Figure 1), new training and nutritional methods were introduced (some in part already during the 2018-2019 season). These methods included training with low CHO availability during most LIT sessions, including long-duration LIT sessions (>4 h) in a fasted state, and days with two MIT sessions on a regular basis. In the 2019-2020 season, the participant performed almost all first sessions of the day in a fasted state and ∼10 days (∼20 sessions) with two MIT sessions during a ∼3-month period in the *preparation period*. However, in the subsequent 2020-2021 season, the participant performed less days with two MIT sessions and less LIT sessions with low CHO but increased the number of LIT sessions >4 h in a fasted state. Coincided with the introduction of these training and nutritional methods, the participant reduced his CHO oxidation during rest, as well as body mass (from ∼75 to ∼70 kg), and body fat percentage (from ∼17 to ∼10%) over a ∼1-year period. The different challenges, symptoms, and possible contributing factors associated with the unexpected underperformance are described in Table 1, whereas detailed training data for the above-mentioned seasons are presented in Table 2.

**TABLE 1:** Different challenges, symptoms, and possible contributing factors associated with unexpected underperformance over two seasons in a world-class male cross-country skier.

Challenges	Symptoms	Contributing factors
• Underperformance	• Lack of energy during training and competitions	• Lack of planned and systematic training periodization
• Unstable performance	• Early fatigue during competitions	• Lack of systematic monitoring, physiological testing, and accurate intensity control
• Perceived imbalance between training and recovery	• Abnormal heart rate responses during training	• Too much training with low CHO availability without careful periodization and monitoring
• Lack of self-efficacy and confidence in the training process	• Lack of self-efficacy	• Too “extreme” regime of training with low CHO including long-duration LIT sessions
• Use of too high cycle rate in the main sub-techniques during both classical and skating	• Lack of motivation to compete	• Days with two MIT sessions without accurate intensity control • Lack of systematic technique training and follow-up by coaches during training on a day-to-day basis
• Too high cycle rate and low “gears” which were effective during LIT but not in competitions	• High mental pressure (perceived mismatch between goals and results)	• Too much accumulated stress over 2-3 seasons

LIT, low-intensity training; MIT, moderate-intensity training; HIT, high-intensity training; CHO, carbohydrate.

**TABLE 2:** Annual training data over two seasons of unexpected underperformance and the season returning to world-class level in a male cross-country skier.

	Unexpected underperformance		Return to world-class level
Season	2019-2020	2020-2021	2021-2022
Total training volume (h)	991	928	990
Total training sessions	527	510	564
Days of sickness	16	20	0
Training forms			
Endurance (h)	937	847	918
Strength (h)	41	62	55
Speed (h)	13	19	17
Training mode			
Specific (h)	654	537	693
Non-specific	283	310	225
Specific/non-specific (%)	70/30	63/37	75/25
Endurance training			
LIT (h)	851	783	839
MIT (h)	54	46	57
HIT (h)	42	18	22
LIT/MIT/HIT (%)	91/6/3	92/6/2	91/6/3
Endurance training			
LIT (sessions)	509	478	547
MIT (sessions)	79	69	76
HIT (sessions)	46	29	33
LIT/MIT/HIT (%)	80/13/7	82/12/6	83/12/5
Low-intensity training			
Warm-up and cool-down (h)	139	110	128
<50 min duration (h)	19	10	13
50–90 min duration (h)	142	142	177
90–150 min duration (h)	308	286	349
>150 min duration (h)	243	235	173
Training methods			
Double-session days (MIT/HIT)	12	5	0
>4 h LIT low CHO (sessions)	12	17	0
Low CHO availability (sessions)	∼70	∼20^a^	0

^a^ Several long-duration sessions in a fasted state.

LIT, low-intensity training; MIT, moderate-intensity training; HIT, high-intensity training; CHO, carbohydrate.

### Returning to sustainable world-class level

After ending the 2020-2021 season, the participant completed 7 weeks with easy, non-systematic training. During this period, detailed analyses of the situation, detection of possible contributing factors to the underperformance, and development of a holistic approach (“treatment”) to the participants training and recovery process were conducted by the support team. Thereafter, different training, nutritional, physiological, and technical interventions were introduced (see Table 3 for detailed description). The goal of the training prescribed was to mitigate risks, improve the load-recovery balance, and thereby ensure better consistency in the training process. Altogether, the participant quickly started to respond positively to the different interventions which resulted in optimal preparations and the return to sustainable world-class level in the following season. Examples of two high-load training weeks in the *preparation period* during one season with underperformance and the season returning to world-class level are shown in Table 4. During the 2021-2022 season, the participant achieved one win and one podium place in World-cup competitions, as well as 3 wins in important National- and Scandinavian-cup qualification competitions. It should also be mentioned that the participant lost his place on the national team and corresponding resources after the 2020-2021 season, and that the return to world-class level was achieved by use of a private waxer. Despite not being a part of the national team, the participant was allowed to take part in training camps with the national team in the preparations to the 2021-2022 season.

**TABLE 3:** Different training, nutritional, physiological, and technical interventions in the holistic approach of returning from unexpected underperformance to sustainable world-class level in a male cross-country skier.

Training	Nutritional
• Introduction of a planned, systematic micro-cycle periodization (easy-, moderate-, and heavy-load-weeks)	• Exclusion of all training with low CHO availability
• Better intensity control by use of blood lactate measurements during training (and reduced intensity during MIT sessions)	• Increased CHO intake during and between training sessions. CHO intake matched with the goal of the session to ensure optimal performance (30 g/h in <60 min sessions, 60 g/h 60–180 min sessions, and 90 g/h during some demanding MIT/HIT sessions)
• More “conservative” training process by excluding all days with two MIT sessions and long-duration LIT sessions (not exceeding 3 h duration)	• Continuous blood glucose monitoring during both rest and exercise
• Assistant coach present in day-to-day training	• Increased evening protein intake (∼20 g) to enhance glycogen resyntheses

Physiological	Technical
• Regular physiological testing (mainly blood lactate profiling in running) in each meso-cycle	• Increased emphasize on strength/power in ski-specific exercises mimicking the technical patterns in cross-country skiing
• Daily monitoring of resting heart rate and body mass	• Focus on reducing cycle rate and increasing cycle length in the main sub-techniques during both classical and skating
• Standardized training sessions with physiological measurements (heart rate and blood lactate measurements)	• Technical focus during all training sessions
	• Video-feedback during key sessions on ski/roller ski each week with clearly defined technical assignments

LIT, low-intensity training; MIT, moderate-intensity training; HIT, high-intensity training; CHO, carbohydrate.

**TABLE 4:** Example of two high-load training weeks during the preparation period in one of the seasons associated with unexpected underperformance and the season returning to world-class level in a male cross-country skier.

	Underperformance (2019-2020)	Return to world-class level (2021-2022)
Monday	AM: 3 h LIT roller-ski classical PM: 2 h LIT running	AM: 3 h LIT running PM: 2 h LIT roller-ski classical
Tuesday	AM: 4 h LIT session running (*fasted state*) PM: Rest	AM: 3 h LIT roller-ski classical PM: 1.5 h speed session roller-ski skating (including LIT warm-up and cool-down)
Wednesday	AM: 2 h (6 × 10 min) MIT interval roller-ski skating uphill (including LIT warm-up and cool-down) PM: 1.5 h (30 min continues) MIT/HIT roller-ski classical double-poling (including LIT warm-up and cool-down)	AM: 2 h (3 × 24 min) MIT interval roller-ski classical (including LIT warm-up and cool-down)—*with coach and video-feedback* PM: 1.5 h strength training (including LIT warm-up and cool-down)
Thursday	AM: 3 h LIT roller-ski classical PM: 2 h LIT running	AM: 3 h LIT running PM: 2 h LIT roller-ski classical
Friday	AM: 4 h LIT roller-ski classical (*fasted state*) PM: Rest	AM: 2.5 h LIT roller-ski skating (including .5 h speed training) PM: 1.5 h LIT running
Saturday	AM: AM: 2 h (6 × 8 min) MIT interval roller-ski classic (including LIT warm-up and cool-down) PM: 2 h LIT roller-ski classical	AM: 2 h (6 × 10 min) MIT interval roller-ski skating (including LIT warm-up and cool-down)—*with coach and video-feedback* PM: 1 h strength training (including LIT warm-up and cool-down)
Sunday	AM: 2.5 h LIT running PM: PM: 1.5 h speed session roller-ski skating (including LIT warm-up and cool-down)	AM: 3 h LIT roller-ski classical (mainly double-poling) PM: 2 h LIT roller-ski skating
Total	LIT (sessions/hours): 8/25 MIT (sessions/hours): 2/2 HIT (sessions/hours): 1/0.5 Endurance training load (TRIMP): 1890 Strength (sessions/hours): 0/0 Speed (sessions/hours): 1/1 Total (sessions/hours): 12/28.5	LIT (sessions/hours): 9/25 MIT (sessions/hours): 2/2.5 HIT (sessions/hours): 0/0 Endurance training load (TRIMP): 1860 Strength (sessions/hours): 2/1.5 Speed (sessions/hours): 2/1.5 Total (sessions/hours): 14/30.5

LIT, low-intensity training; MIT, moderate-intensity training; HIT, high-intensity training; TRIMP, training impulse score.

**FIGURE 2:**
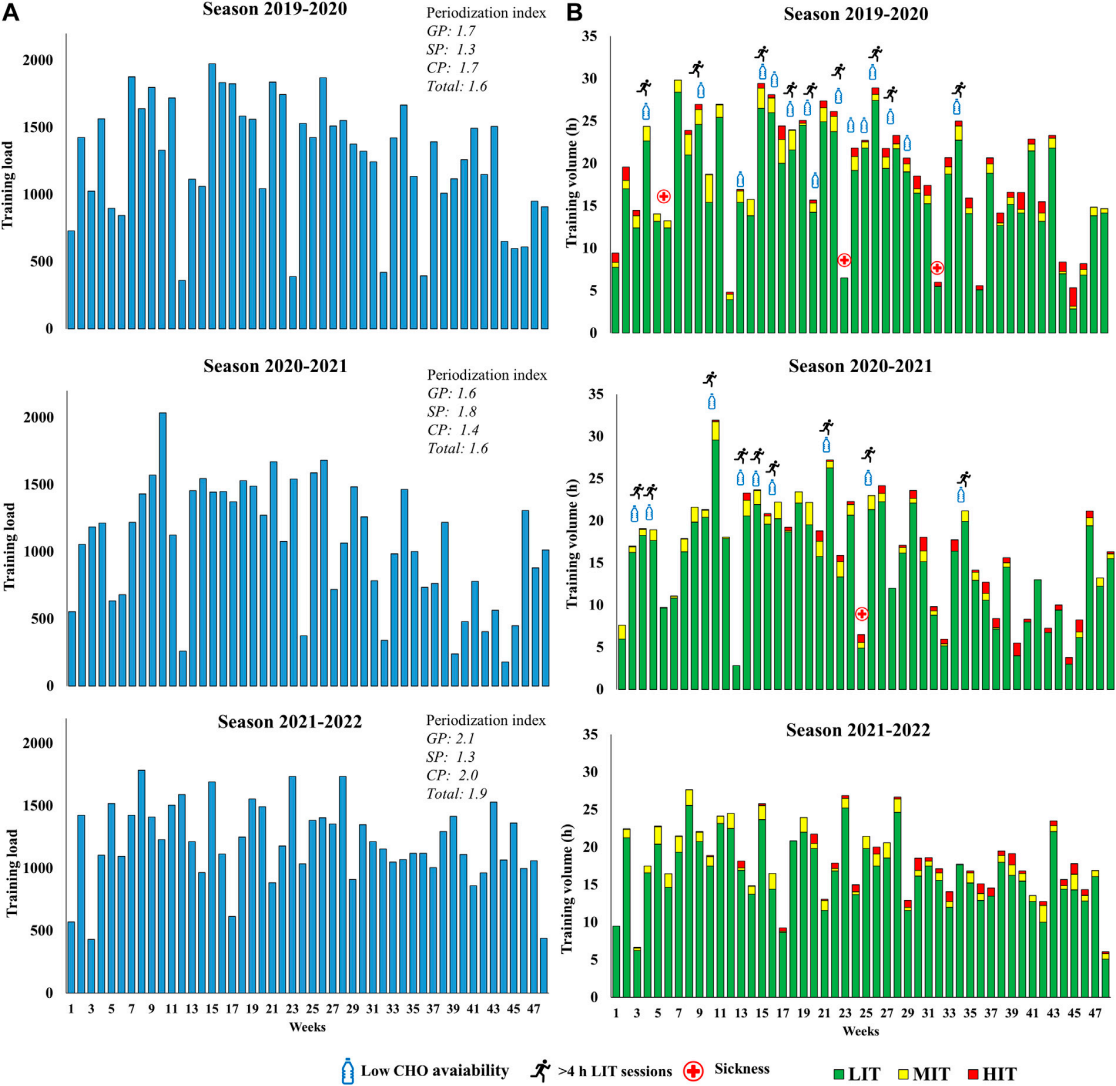
Weekly **(A)** training load and **(B)** endurance training volume distributed as low- (LIT), moderate- (MIT) and high-intensity training (HIT) over two seasons of unexpected underperformance (2019-2020 and 2020-2021) and the season returning to world-class level (2021-2022) in a male cross-country skier. GP indicates general preparation period; SP, specific preparation period; CP, competition period.

**FIGURE 3:**
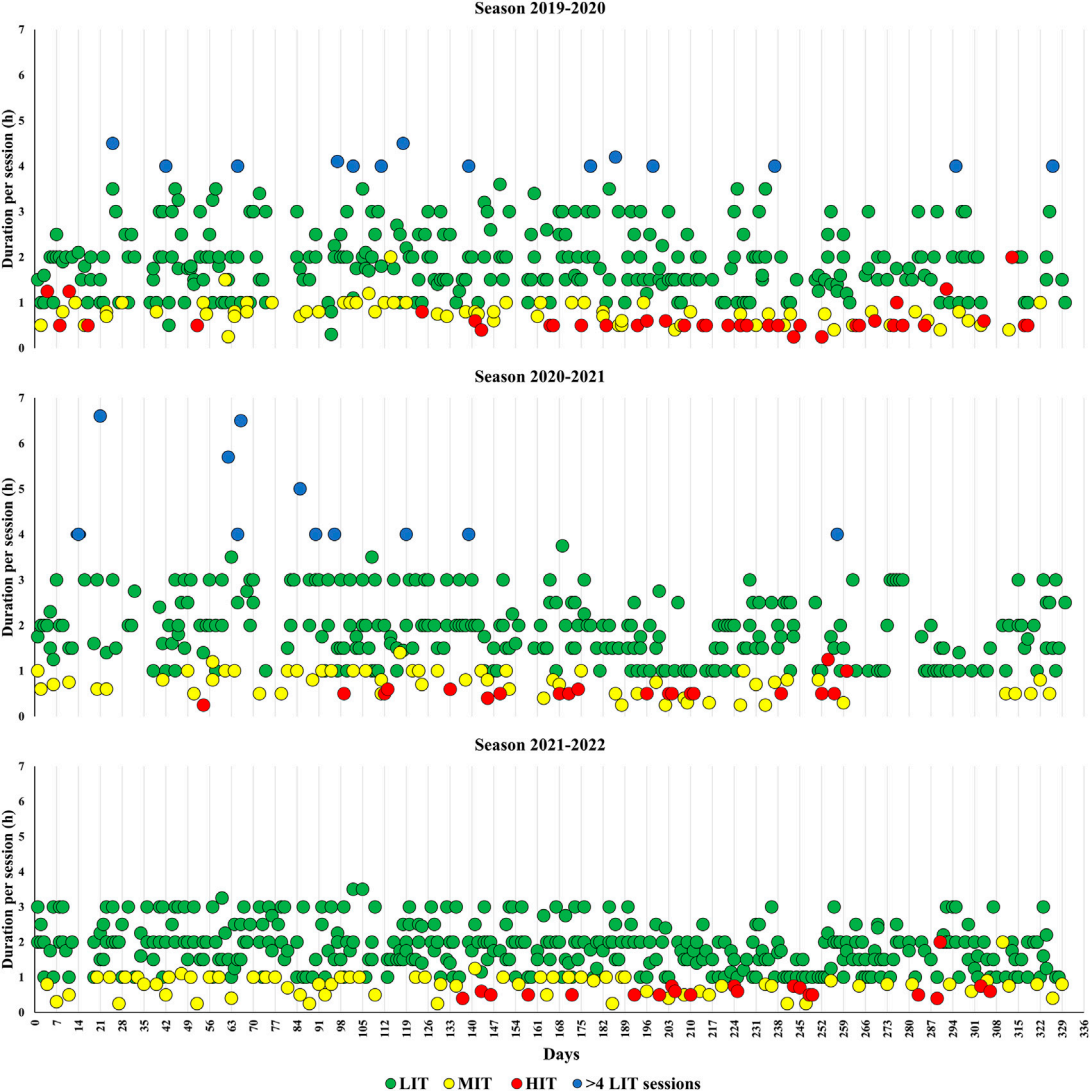
The duration of low- (LIT), moderate- (MIT) and high-intensity training (HIT) sessions over two seasons of unexpected underperformance (2019-2020 and 2020-2021) and the season returning to world-class level in a male cross-country skier. *LIT in connection with warm-up and cool-down are not included.

Weekly training loads and endurance training volumes, as well as the duration of different endurance training sessions in the seasons with underperformance and the season returning to world-class level are displayed in Figures 2, 3 . Although the training intensity distribution remained similar as in the two preceding seasons with underperformance, total training volume and endurance training volume increased by 7% and 8% from the 2020-2021 season (explained by ending the latter season already in January). However, there was an increase in the volume of specific endurance training and a corresponding decrease in the volume of non-specific training in the 2021-2022 season compared to the 2019-2020 and 2020-2021 seasons (Table 2). This was mostly explained by a larger amount of running in connection with the long-duration LIT sessions in the 2019-2020 and 2020-2021 seasons. Moreover, the *periodization index* indicated a somewhat larger difference between high and low micro-cycle loads in the 2021-2022 season compared to the 2019-2020 and 2020-2021 seasons. Lastly, days of sickness were reduced in the 2021-2022 season compared to the two seasons with underperformance.

Coincided with the above-mentioned interventions, the participant improved his blood lactate profiling with increased speed_@4mmol_ in the *general preparation period* which remained at this level in the *specific preparation* and *competition period* (Table 5). Further, while the participants body mass remained relatively similar (∼70 kg), there was an increase in body fat percentage (from ∼10 to ∼14%) in the season returning to world-class level.

**TABLE 5:** Blood lactate profiling in running at different time points in the season returning to world-class level in a male cross-country skier.

	GP1	GP2	SP1	SP2	CP
Body mass (kg)	70.1	71.0	71.0	70.4	70.0
Perceived readiness (1–10)	7	8	7	8	8
Day shape (1–10)	8	6	6	8	7
Speed_@4mmol_ (km·h^−1^)	12.5	12.7	13.1	12.9	13.0
HR_@4mmol_ (beats·min^−1^)	184	187	187	184	189
RPE_@4mmol_ (6–20)	14.1	12.6	12.7	13.0	14.1

GP1, general preparation period test 1; GP2, general preparation period test 2; SP1, specific preparation period test 1; SP2, specific preparation period test 2; CP, competition period test; _@4mmol_, 4 mmol L^−1^ blood lactate concentration HR, heart rate; RPE, rating of perceived exertion.

## Discussion

The purpose of this case study was to determine the main factors associated with unexpected underperformance, and prospectively describe the holistic approach of returning to sustainable world-class level in a male cross-country skier. The main findings were that: 1) following two seasons of unexpected underperformance, the possible contributing factors were identified: lack of training periodization, limited monitoring and intensity control, particularly in connection with a “extreme” regime of training with low CHO and days including two MIT sessions, as well as a lack of systematic technique training and follow-up by coaches on a daily basis, and 2) consequently, the holistic approach of returning to world-class level included an introduction of a clear micro-cycle periodization, more systematic physiological monitoring and testing, more accurate intensity control, increased CHO intake during and between sessions, as well as increased emphasize on technique training and an assistant coach present during day-to-day training.

### Longitudinal training data leading to a world-class level

Initial analyses of the long-term training data while gradually progressing towards a world-class level were performed to understand the participants training history and to identify the changes done in the 2019-2020 to 2020-2021 seasons which may have contributed to the unexpected underperformance. Here, the participant demonstrated a non-linear progressive increase of 30% in total training volume (772–1,002 h annually) from 21 (early senior athlete) to 28 years of age. The progression in training volume was mainly caused by increased endurance training volume and particularly LIT. These data extend upon previous case studies of endurance athletes (Stellingwerf, 2012; Bourgois et al., 2014; Pinot and Grappe, 2015; Rasdal et al., 2018; Schmitt et al., 2020) including the most decorated female cross-country skier (Solli et al., 2017), emphasizing the importance of high training volumes to reach a world-class level, and that this may successfully be achieved by adopting a non-linear annual volume progression. The observed progression in training volume prior to the seasons of underperformance in the present case may have allowed the participant to tolerate and respond positively to increased training loads while mitigating the risk of maladaptation, sickness, and injury. Interestingly, the increase in endurance training volume was mainly specific (skiing and roller-skiing), whereas the volume of non-specific training (almost exclusively running) remained relatively similar, suggesting increased emphasize on targeting the sport-specific demands of cross-country skiing throughout the career. This was further confirmed by the participant during interviews, and due to the same reason, the participant also increased the volume of speed training with a five-fold to close the gap between the sport-specific demands and the participants limitation in “high-speed capacities”. Altogether, these longitudinal data suggests that progression in endurance training volume in general, and within sport-specific modes in particular, likely contributed to achieving a world-class level in cross-country skiing. Further, these data provide an important basis for analyzing the subsequent changes that may have contributed to the unexpected underperformance in the two following seasons.

### Factors associated with unexpected underperformance

Although the total training volume and intensity distribution remained relatively similar in the two seasons of underperformance as in the preceding seasons, new training and nutritional methods were introduced. These methods included a rather “extreme” regime of training with low CHO availability and days with two MIT sessions without sufficient intensity control. Clearly, these methods induced additional stress-loads to the training process that over time probably contributed to the development of unexpected underperformance (with or without the diagnosis of RED-S and/or OTS). Therefore, it may be speculated that the training and nutritional methods implemented resulted in an accumulation of stressors peaking in the 2020-2021 season. This hypothesis is further supported by the case study of (Solli et al., 2020), where the periods of underperformance and corresponding training and non-training stressors accumulated over several seasons before reaching a negative turning point in performance. In addition to the above-mentioned methods, the lack of a planned and structured micro-cycle periodization (load structure) was detected as a possible contributing factor to underperformance. However, despite that the developed *periodization index* only revealed small differences, the micro-cycle load structures during the seasons of underperformance were characterized by accumulation of high stress-loads until “forced” easy-load cycles were needed. Thus, these cycles were included due to sickness and/or fatigue following cycles rather than a strategic and planned periodization approach. Accordingly, this led to an unsustainable training process characterized by a lack of consistency. Therefore, we cannot conclude that the introduction of training with low CHO availability and/or days with two MIT sessions *per se* led to the underperformance (with or without the diagnosis of RED-S and/or OTS). However, the introduction of such methods in a training process already characterized by high stress-loads without careful periodization, monitoring, and intensity control are likely contributing factors.

Training with low CHO were implemented during most LIT sessions (particularly 2019-2020 season), including long duration LIT sessions in running (particularly 2020-2021 season). These changes were reflected by a larger proportion of the LIT volume performed as long sessions (∼4–6 h duration) and a larger proportion of the endurance training volume performed as running. The rationales for implementing training with low CHO provided by the participant during interviews were complex. On one hand, the participant had experienced periods with CHO depletion (“hitting the wall”) during competitions and, thus, training with low CHO availability was suggested as a method to enhance fat oxidation both during rest and training/competitions. On the other hand, training with low CHO availability had the intention of augmenting adaptations from the high endurance training loads, and thereby enhance the participants performance level. Indeed, there exists scientific evidence suggesting that training with low CHO availability have the potential to increase molecular signaling associated with skeletal muscle adaptations (Burke, 2010; Bartlett et al., 2015). However, a recent meta-analysis of nine studies demonstrated no performance adaptations of periodized CHO availability (Gejl and Nybo, 2021) suggesting that this method does not enhance performance beyond training with high CHO availability. Although training sessions are initiated with adequate muscle glycogen stores, it could be argued that the high training loads of endurance athletes (including twice-a-day sessions) already induces sufficient “train low” molecular signaling (Podlogar and Wallis, 2022). Rather, such methods may concurrently increase the athletes stress-recovery ratio and the risk of developing RED-S and/or OTS (Jeukendrup, 2017; Stellingwerff et al., 2021). Therefore, considering the different challenges, symptoms, and rather “extreme” nutritional regime followed, the participant may have developed a state of RED-S and/or OTS. However, the participant was not diagnosed with RED-S and blood markers of RED-S were considered within normal ranges although a reduction in body fat percentage was seen during the two seasons of underperformance. Moreover, considering that no single and valid test for OTS diagnosis exists and the long time required for performance restoration (Meeusen et al., 2013), a full-blown OTS diagnosis might be excluded. However, the period of underperformance in this case was relatively long (∼2 seasons), and taken into account the complex etiology of OTS and RED-S (Stellingwerff et al., 2021), it may be difficult to separate and fully confirm these conditions in the present case.

Moreover, the participant implemented days with two MIT sessions in the training process and particularly during the first season of underperformance (2019-2020 season). Although not yet scientifically investigated, anecdotal evidence of positive effects of adopting days with two MIT sessions (“double-threshold”) exists among elite to world-class endurance athletes. In the present case, this was implemented as a method to progress the volume of MIT with the intention of augmenting endurance adaptations. This was further reflected by an increase in both the number of sessions and volume of MIT in the 2019-2020 season (in part already during the 2018-2019 season) compared to the preceding seasons. However, although days with two MIT sessions may successfully be adopted by endurance athletes, these days were likely too demanding in the present case considering the already high stress-loads due to a lack of training periodization and intensity control, as well as the “extreme” low CHO availability regime.

The seasons of underperformance were further characterized by a lack of systematic technique training and follow-up by coaches on a day-to-day basis outside training camps. The underperformance was coincided by technical patterns characterized by too high cycle rates and use of low “gears” in both the skating and classical technique. These technical patterns were probably effective during LIT but not in competitions where the participant clearly lacked ski-specific power to follow when speeds were increased. Although it seems obvious that the lack of systematic technique training contributed to suboptimal technical patterns, it cannot be overlooked that the above-mentioned factors associated with the underperformance contributed to the use of more economical technical solutions during the high volumes of LIT and thereby induced non-effective technical changes during competitions.

Moreover, it cannot be ignored that the lack of follow-up by coaches on a day-to-day basis was a contributing factor to the underperformance. Obviously, signs of underperformance (e.g., increased variations in performance during training) should have been detected earlier and changes in the rather “extreme” training and nutritional regime, including better monitoring and intensity control should have been done much earlier. However, it should also be acknowledged that detecting RED-S and/or OTS at early stages are difficult and valid methods for OTS diagnosis does currently not exist (Meeusen et al., 2013; Stellingwerff et al., 2021). In fact, a recent meta-analysis failed to identify any scientific evidence for detailed changes in performance and corresponding psychological symptoms prior to the onset of OTS diagnosis (Weakley et al., 2022). Taken together, all the above-mentioned contributing factors to the underperformance can to different extents be related to a lack of periodization, systematic monitoring. and intensity control, as well as follow-up by coaches on a daily basis.

### Returning to sustainable world-class level

The process of returning to world-class level included a 7-week period without any systematic training and detailed analyses of the possible factors leading to underperformance together with the support team and other experts in the field. Through this process, several training, nutritional, physiological, and technical interventions were initiated to target each of the above-mentioned possible contributing factors to the underperformance. This process is comparable to the one previously described by (Solli et al., 2020) and (Gustafsson et al., 2008), both emphasizing the importance of a holistic and individualized approach in “treating” long-term underperformance. In the present case, the participants training history and experiences from the seasons leading up to the underperformance were used as a point of departure. Overall, the targeted interventions included introduction of a clear micro-cycle periodization, more systematic physiological monitoring and testing, more accurate intensity control, increased CHO intake during and between sessions, as well as increased emphasize on technique training and an assistant coach present during training on a daily basis.

The introduced micro-cycle periodization using easy-, moderate-, and heavy-load-weeks were considered important to allow adequate recovery and subsequent adaptations, as well as providing a system for decision making and load adjustments. On average, the easy-, moderate-, and heavy-load-cycles were planned with 15, 20, and 25 h of training, with a progression to 25 and 30 h, in the moderate- and heavy-load weeks, respectively, throughout the *preparation period*. While these volumes were planned, it should be emphasized that they were continuously adjusted according to how the participant adapted. These changes in micro-cycle periodization were in part reflected by the greater *periodization index* and are consistent with previous studies emphasizing the importance of training periodization to optimize adaptations from endurance training (Issurin, 2010). The typical training week consisted, on average, of two MIT/HIT sessions (the majority MIT sessions), two strength sessions, two speed sessions, and the remaining LIT including 2 days of exclusively LIT (typically ∼3 + ∼2 h sessions). Specific training (roller-skiing and skiing) and the corresponding training quality of each session was prioritized, and the long-duration LIT sessions in running excluded from the training process (no LIT sessions above ∼3 h). These changes were reflected by the categorization of different types of LIT and the increased volume of specific endurance training in the season returning to world-class level.

Moreover, the introduction of more systematic physiological testing was important for controlling the training process. In comparison, the seasons associated with underperformance were characterized by more unsystematic use of testing which may have prevented the ability to detect signs of underperformance at an earlier stage. In the return to world-class level, the participant increased speed_@4mmol_ during the *general preparation period* which remained at this level in the *specific preparation* and *competition period*. The lack of further improvement in speed_@4mmol_ after the *general preparation period* was most likely explained by more emphasize on developing ski-specific capacities in the *specific preparation and/or competition period*. Therefore, it could be argued that treadmill roller-ski tests may have served the purpose better, but the use of treadmill running tests have the advantage of decoupling changes in technique and thereby providing a more valid assessment of the actual physiological responses. Moreover, the inclusion of blood lactate profiling in running was not to monitor the performance progression *per se* but rather use the tests as a “control system” and decision-making support.

The exclusion of training with low CHO availability, and particularly the long-duration LIT sessions in a fasted state most likely restored the participants energy levels during training and competitions. Considering the symptoms and possible contributing factors to the unexpected underperformance, the nutritional changes were most likely crucial in the process of returning to a world-class level. Herein, CHO intake during and between sessions followed the recommended nutritional guidelines for endurance athletes (Burke et al., 2011; Podlogar and Wallis, 2022). These guidelines were matched with the fuel needs and muscle glycogen restoration of each session (30 g/h in <60 min sessions, 60 g/h 60–180 min sessions, and 90 g/h during demanding MIT sessions of fructose-glucose-based CHO mixtures), as well as a daily CHO intake of 8–12 g kg/day depending on the training loads. Continuous blood glucose monitoring was used to increase the participants understanding of optimal CHO nutrition although it was experienced that such monitoring was most useful in a non-exercise context (i.e., rest and over nights), possibly due to the complex mechanisms of blood glucose regulations during exercise (Podlogar and Wallis, 2022). Further, the increased evening protein intake was introduced as a method to enhance glycogen resyntheses and particularly in periods with high training loads and fueling requirements (Podlogar and Wallis, 2022). Likely, this contributed to a more sustainable health through increased energy availability and particularly CHO availability, and thereby more positive adaptations to the high endurance training loads in the season returning to world-class level.

Moreover, the return to world-class level excluded the use of days with two MIT sessions. Although this method may have positive effects in other contexts it was considered too demanding considering the possible contributing factors to underperformance. Coincidingly with the above-mentioned introduction of better monitoring an intensity control, the MIT sessions were performed with more systematic use of heart rate recordings and blood lactate measurements. These changes may have been important for better differentiating MIT from typical HIT sessions and thereby reducing the risk-benefit ratio.

Increased emphasize on technical training were among the key interventions during the return to world-class level and included clear technical assignments. The overall goal was to reduce cycle rate and increase cycle length and strength/power in the main sub-techniques, and thereby increase the propulsive forces and subsequent recovery times in both the pole and leg push-off. Interestingly, the sub-optimal technical patterns developed over the period with underperformance were comparable to those described by (Solli et al., 2020), suggesting that sub-optimal technical patterns may coincide with periods of underperformance in endurance athletes.

Lastly, having the assistant coach present during key session in the return to world-class level were regarded as positive by the participant by increasing the training quality through organizing, observing, discussing, ensuring intensity control, and providing video-feedback on technique. Together, increased present of a coach during day-to-day training likely enhanced the training quality of each single session and thereby the participants performance progression. It should also be acknowledged that the accumulated underperformance, peaking in the 2020-2021 season, increased the participant’s understanding of that major changes were required, and he was more open to input and corresponding changes in the training process during the return to world-class level. While the introduced changes likely had independent influence on the performance progression, increased openness and a strong coach-athlete relationship may also have contributed to a greater belief in the training process. In this connection, adaptations to training are not solely dependent on the physiological/mechanical stimulus, but also associated psycho-emotional considerations such as the athletes understanding and belief in the training plan, as well as a sense of purpose and “ownership” to the training process (Kiely, 2018).

### Practical applications

The present case study provides important knowledge to coaches and athletes in endurance sports and particularly in cases of long-term underperformance. First, as also shown previously, this study indicates that a holistic approach in “treating” underperformance is beneficial, by targeting those factors of the training and recovery process seen as contributing factors. Herein, using sufficient time to detect the contributing factors to underperformance, as well as the subsequent interventions for performance restoration seems crucial. Second, systematically planned and implemented micro-cycle periodization seems beneficial in such a process, with the developed *periodization index* serving as a relevant indicator of periodization that can be adopted to other micro- and meso-cycle durations than those used in the present study. Third, a training philosophy based on mitigating risks (reducing the risk-benefit ratio) by performing the majority of training sessions with “control” seems beneficial. This may be important not only for underperforming endurance athletes, but endurance athletes in general. Rather, increased emphasize should be put towards optimizing the training quality of each single session (physically, mentally, and technically) and thereby ensuring a more sustainable and consistent training process. Forth, systematically, and frequent physiological testing (or standardized training sessions) may serve as important tools for detecting adaptations/maladaptation’s, as well as providing decision support for load adjustments (e.g., green light = continue as planned, yellow light = minor load adjustments, red light = large load adjustments). However, care should be taken in the interpretation of the present data, as no cause-effect relationships can be established, and generalization of the findings therefore limited. The authors are also aware that the study may be limited by confirmation and survivorship bias ignoring the fact that other endurance athletes with comparable challenges may have undergone much of the same interventions without achieving the same level of performance restoration. However, taken together, the present case study may serve as theoretical framework for future studies and practical work with underperforming endurance athletes (with or without the diagnosis of RED-S and/or OTS).

## Conclusion

This case study describes the main factors leading to unexpected underperformance, in addition to providing unique insights into the corresponding process of returning to sustainable world-class level in a male cross-country skier. The seasons of underperformance were characterized by a lack of systematic periodization and monitoring, as well as the implementation of a too “extreme” regime of training with low CHO availability leading to unsustainable training loads. Accordingly, the participant’s return to world-class level targeted these possible reasons for underperformance by a clear micro-cycle periodization, more systematic physiological monitoring and testing, optimized CHO intake, as well as increased emphasize on intensity control and technical development through closer follow up during daily training.

## Data Availability

The raw data supporting the conclusion of this article will be made available by the authors, without undue reservation.
